# Retinal and choroidal microvascular assessment of children receiving recombinant growth hormone therapy: Study design: a prospective observational comparative study

**DOI:** 10.1186/s40942-024-00610-z

**Published:** 2024-11-22

**Authors:** Ismail Omar, Yousra Samir Fadle, Noura M. Ibrahim El Bakry

**Affiliations:** 1 Ophthalmology Department, Faculty of Medicine, Minia University, El-Minya, Egypt; 2 Pediatric Departement, Faculty of Medicine, Minia University, El-Minya, Egypt

**Keywords:** Growth hormone deficiency, Hormone therapy, Retinal microvasculature, OCTA

## Abstract

**Background:**

The purpose of this study is to evaluate the retinal and choroidal microvascular state in children with congenital isolated growth hormone deficiency (IGHD) and determine the effect of recombinant human growth hormone treatment on these structures compared with healthy controls.

**Methods:**

The study included children with IGHD under recombinant human GH treatment as group one and another group of healthy controls. Both groups were examined using optical coherence tomography angiography (OCTA). Data concerning superficial capillary plexus (SCP), deep capillary plexus (DCP), choriocapillaris (CC), and retinal thickness were recorded.

**Results:**

The study included two equal groups of 30 individuals. Both groups had no statistically significant differences in age, gender, weight, or spherical equivalent. However, subjects of group II were taller than those of group I (*p* = 0.011). OCTA images of the SCP, DCP, and CC vessel density revealed statistically non-significant differences between the two groups.

**Conclusion:**

Children receiving recombinant growth hormone therapy showed no changes in the retinal and choroidal microvasculature or macular thickness.

**Trial registration number:**

1094/03/2024 by Minia University Faculty of Medicine Institutional Review Board. Another registration number is UMIN000055654.

## Background

Growth hormone deficiency (GHD) is a condition with clinical, metabolic, and biochemical abnormalities in which the production of growth hormone (GH) and growth factors is decreased [1].

Congenital isolated growth hormone deficiency (IGHD) is defined as childhood growth retardation because of decreased GH [2]. The demonstration of poor linear growth, delayed skeletal age, and peak levels of GH (< 7ng/ml) in each of the two provocative tests is compatible with diagnosing GH deficiency [3, 4].

Somatotropes of the pituitary gland are responsible for GH synthesis and secretion. The hypothalamic hormones regulate GH secretion in a pulsatile fashion by the alternating secretion of GH-releasing hormone (GHRH) and somatostatin [5, 6].

GH has been shown to have angiogenic effects. Retinal cells are a domain of local growth factors, including insulin-like growth factor-1 (IGF-1), which also has a role in retinal growth and development in fetal life [7, 8].

Although the mechanisms by which GH affects angiogenesis are unknown, GH has a role in retinal angiogenesis and its influence on neural development [9].

In clinical studies, the effect of deficiency of GH on retinal vascularization was demonstrated as decreased vascular branching points, assessed by a semi-quantitative analysis of fundus photography [10, 11].

Optical coherence tomography angiography (OCTA) provides a three-dimensional visualization of the perfused retinal and choroidal vasculature [12].

OCTA visualizes the structure and blood flow in the retina and choroid, allowing examination of the different capillary networks of the retina, with vessel diameters around 8 μm [13]. OCTA also assesses the temporal changes of the OCT signal. Repeated OCT images (B-Scans) from the same point on the retina show temporal signal changes due to moving particles (such as erythrocytes flowing through vessels), creating an image contrast between the perfused vessels and the surrounding static tissues. Through dense volume scans, it is possible to obtain OCTA images like fluorescence angiography images, which are the clinical gold standard but require dye injection [14].

### Aim of the work

This study aimed to evaluate the retinal and choroidal microvascular state in children with congenital isolated growth hormone deficiency (IGHD) and determine the effect of recombinant human GH treatment on these structures compared with healthy controls.

## Subjects and methods

It is a prospective cross-sectional comparative study. This study was approved and conducted in accordance with the tenets of the Declaration of Helsinki, and written informed consent was obtained from all subjects’ legal guardians. The local institutional review board and ethics committee of the faculty of medicine of Minia University approved the study protocol (1094/03/2024).

The study comprised two groups of children: the first group of patients with growth hormone deficiency receiving recombinant growth hormone, and the second group was normal controls. Each group had 30 subjects. The patients were recruited from the pediatric endocrinology unit of the pediatrics department of Minia University Hospital. Ophthalmic examination was done in the ophthalmology department of the same hospital.

### Inclusion criteria

Enrollment of the patients in the study was based on age equal to or less than 16 years old, and patients were not receiving any therapy other than recombinant GH for group 1, clear ocular media, and no history of chronic ocular or systemic diseases.

### Exclusion criteria

The patients were excluded from the study if they had a history of preterm or small-for-gestational-age birth, a diagnosis of cardiovascular disease, thyroid disease, hepatic or renal disorder, obesity, current hypertension, or the presence of chromosomal abnormalities. Also, the study did not include children with high myopia, hyperopia, amblyopia, or ocular disease.

### Physical examination and investigations

The children of the two groups had a thorough physical examination. The anthropometric measurements of the weight and the height were recorded. The bone age was evaluated using the *Greulich& Pyle atlas.* Routine investigations like complete blood count, thyroid function test, and serum tissue transglutaminase antibodies were tested for all study patients. Diagnosis of IGHD was established by the clinical and biochemical criteria of the American Association of Pediatrics (AAP). We have chosen isolated growth hormone deficiency cases as they all had no other pituitary hormone deficiencies and had normal MRI pituitary glands. Patients of group 1 were receiving recombinant human GH treatment of variable doses and durations.

### Ophthalmologic examination

The participants underwent a complete ophthalmologic examination starting from history and then examination of the anterior segment with slit-lamp biomicroscopy, fundus examination, intraocular pressure measurement, and state of refraction. Afterward, an assessment with optical coherence tomography angiography (OCTA) was performed on all study participants by the same experienced examiner. The right eye was the targeted eye for all participants. The 6 × 6 mm scans were used to evaluate the vessel density of the superficial capillary plexus (SCP), deep capillary plexus (DCP), and choriocapillaris (CC). Each previously mentioned parameter was tested in the whole, foveal, and parafoveal zones. The vessel densities of the superior, inferior, temporal, and nasal quadrants were recorded for the parafoveal zone. Furthermore, the OCTA thickness of the different zones was obtained. These recordings were obtained from the instrument’s display image, which was automatically calculated by the built-in software.

### Statistical analysis

Data obtained from the study were recorded and statistically analyzed using the Statistical Package for the Social Sciences for Windows(version 21, SPSS, Chicago, IL, USA). Kolmogorov–Smirnov test was used for normality testing. Quantitative variables were expressed as mean ± standard deviation. Differences between groups were assessed using the independent samples t-test. The statistical level of significance was set to *P* < 0.05.

## Results

The study participants as a whole were 60 patients divided into two equal groups. Table 1 presents the demographic and refractive data of the two groups. Most of the children were in the late childhood stage, and most of them were male patients. The refractive state of the two groups was near emmetropia. Statistically, the groups had no significant differences in age, gender, weight, and spherical equivalent. However, there was a statistically significant height difference, as the subjects of Group II were taller than those of Group I (*p* = 0.011).

**Fig. 1:**
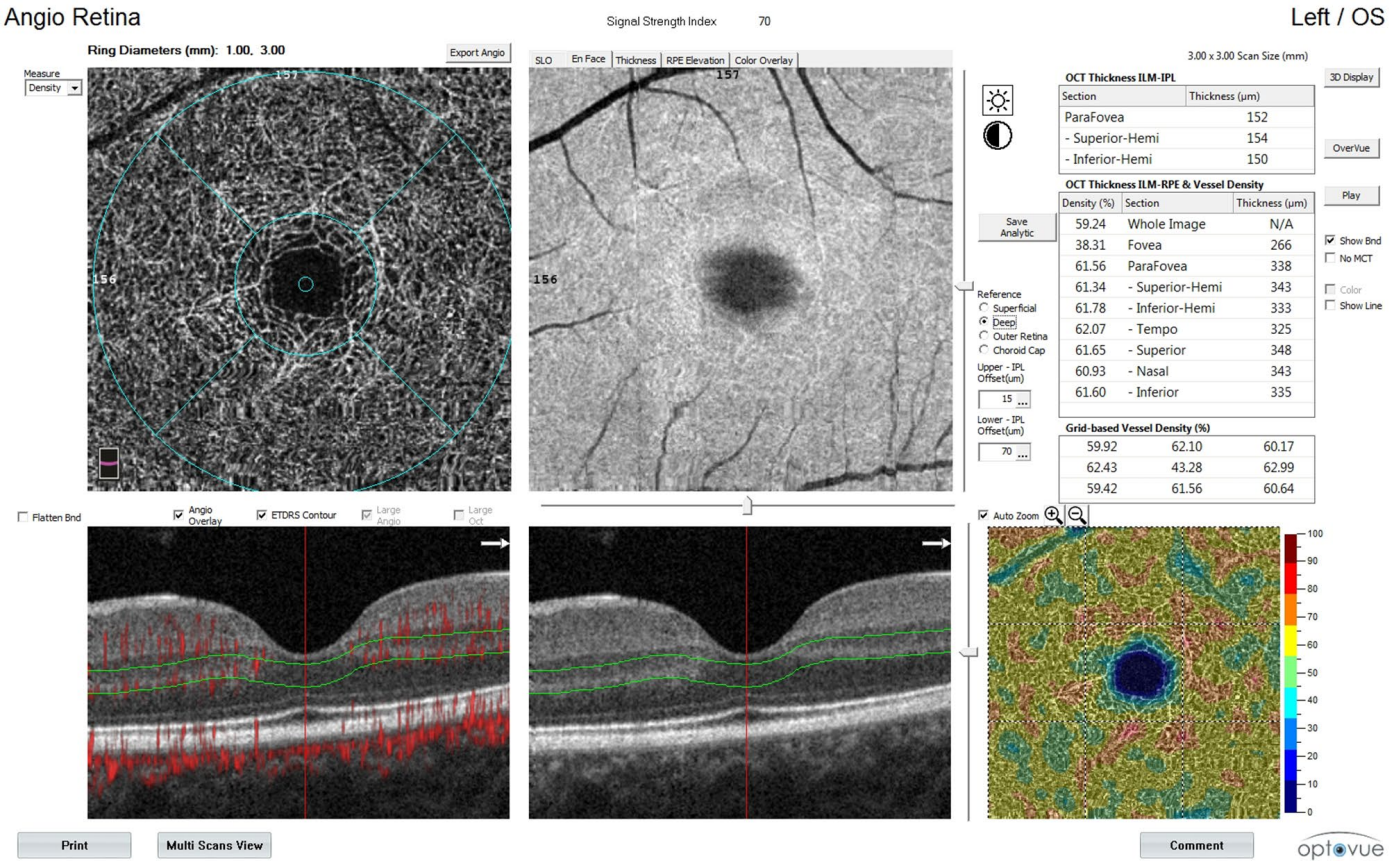
OCT angiography image of the vessel densities at the deep capillary plexus

The data were obtained from the OCTA images of the SCP, DCP, and CC vessel density. These data were collected from a table on the top right side of the OCTA display (Fig. 1). The obtained measurements were analyzed and recorded in Tables 2 and 3, and 4 for SCP, DCP, and CC, respectively.

The analysis of the mean VDs of the whole image in DCP and CC showed that they were more in group II than in group I, but the VDs of SCP were approximately the same in the two groups. The foveal VDs of SCP, DCP, and CC were higher in group I.

The parafoveal VDs of the SCP were higher in group I, but those of DCP and CC were higher in group II. The parafoveal quadrantic analysis of the vessel densities showed the same results as the total parafoveal VDs analysis.

Studying the OCTA thickness in both groups revealed minimal differences, as shown in Table 5.

Although there were differences between the study and the control groups in many parameters, these differences were not statistically significant.

## Discussion

Growth hormone affects the growth of various body organs and the eyes. It was postulated that growth hormone reaches the eyes through the choroidal vasculature and shares in the retinal development [15]. Many studies showed the effects of GH and GHD on the retina and the optic disc. Still, none of them studied the impact of the recombinant growth hormone therapy on the vessel density of the retinal and choroidal capillary plexuses by the OCTA.

Growth hormone deficiency does not affect only the children’s height but also the health, quality of life, and psychological state of these patients [16]. A study conducted on GHD children to report their anxiety disorders concluded that they had higher generalized anxiety disorder and social anxiety disorder burdens in comparison to healthy children. Therefore, the management of children with GHD requires coordination between mental health professionals and pediatric endocrinologists. So, recombinant GH therapy has been used frequently to treat GHD, not only to increase the children’s height but also to improve their behavior and attitude [17, 18].

As GH treatment is used for long durations, our study was conducted to observe whether GH therapy harms the retinal and choroidal vasculature or not.

The main difference between our studied groups in the anthropometric measurements was that the children with normal growth rates had statistically significant taller height, which was expected. Patients with high errors of refraction were excluded from the study to avoid the effect of external factors on the retinal microvasculature. Most of the children studied in both groups had low errors. Moreover, examination of the patients revealed free fundus examination of any retinal changes.

Analysis of vessel density of the superficial and deep capillary plexuses showed no statistically significant differences between the two groups. Studying the effect of growth hormone therapy on CC showed that the VD in all study zones of group I was not statistically significantly different than group 2.

The thickness of the retina was studied in each zone of each group, and differences between groups were compared. The thickness of the fovea and parafoveal zones revealed no statistically significant differences between the groups. This suggests that GH therapy did not affect the macular thickness in any way, even if taken for long periods, as we had patients on GH therapy for more than three years.

Many researchers have studied the impact of GH deficiency and therapy on the refractive and structural conditions of the eye.

It was reported that GH deficiency had affected the GH-deficient subjects’ axial length and refractive state as their refraction was towards hyperopia with shorter axial length compared to healthy subjects [19].

Moreover, growth hormone treatment effects on the spherical equivalent and the axial length were measured in a previous study. They found non-significant changes between pre-GH therapy and post-therapy measurements [20].

Previously, the effect of growth hormone deficiency on the quantitative measurements of the retinal and choroidal vessel density by OCTA was studied in patients with untreated growth hormone deficiency. This study concluded that IGHD did not affect the retinal microvasculature [21].

However, other researchers reported that IGHD may lead to decreased retinal vasculature and reduced vascular branching points. These studies focused on the major vessels and did not use OCTA [11, 22].

The macular thickness was studied in a comparative study on the IGHD patients’ group versus a control group. The authors of this study found no difference in the macular thickness between the two groups before starting the therapy. After starting the GH treatment, the patients showed no changes in the macular thickness or the retinal vascular tissue [22].

Furthermore, macular thickness was not affected in another earlier study assessing the growth hormone deficiency effect on macular thickness [11].

This was the first study on the effect of GH therapy on the retinal microvasculature; however, only a small sample of cases was studied. Moreover, although weight was similar between the groups, height was different, so body mass index (BMI) was different between the two groups, which might affect the investigated parameters.

## Conclusions

Children receiving recombinant growth hormone therapy showed no changes in the retinal and choroidal microvasculature or macular thickness.

**Table 1:** Demographic and refractive data

	Group I	Group II	*P* value
Age (years)	11.63 ± 2.96	11.29 ± 63	0.21
Gender (Male/Female)%	66.67/33.33	73.33/26.67	0.078
Height (cm)	141.4 ± 11.68	152 ± 11.83	0.011
Weight (kg)	36.21 ± 8.3	36.09 ± 10.15	0.36
Spherical equivalent (D)	-0.067 ± 1.83	-0.074 ± 1.92	0.67
Duration of treatment (months)	24.85 ± 8.127	NA	NA

*cm: centimeters, kg: kilograms, D: diopter

**Table 2:** The vessel density of the superficial capillary plexuses (SCP)

SCP	Group I	Group II	*P* value
Whole image	48.768 ± 3.392	48.519 ± 3.606	0.138
Foveal VD	29.482 ± 6.321	26.124 ± 2.071	0.095
Parafoveal VD	51.280 ± 3.470	50.479 ± 3.999	0.772
Temporal quadrant VD	51.203 ± 3.186	50.542 ± 3.879	0.861
Superior quadrant VD	51.342 ± 4.181	50.482 ± 5.31	0.732
Nasal quadrant VD	50.388 ± 4.505	50.187 ± 5.413	0.693
Inferior quadrant VD	51.754 ± 3.832	50.981 ± 4.626	0.581

*SCP: superficial capillary plexuses, VD: vessel density

**Table 3:** The vessel density of the deep capillary plexuses (DCP)

DCP	Group I	Group II	*P* value
Whole image	55.078 ± 5.862	58.637 ± 3.224	0.61
Foveal VD	32.457 ± 9.896	26.383 ± 7.349	0.086
Parafoveal VD	59.407 ± 4.613	63.390 ± 4.292	0.068
Temporal quadrant VD	59.275 ± 4.212	62.364 ± 4.981	0.071
Superior quadrant VD	59.521 ± 4.848	61.645 ± 6.372	0.082
Nasal quadrant VD	59.798 ± 5.839	61.34 ± 6.18	0.093
Inferior quadrant VD	58.638 ± 5.012	59.726 ± 3.182	0.062

*DCP: deep capillary plexuses, VD: vessel density

**Table 4:** The vessel density of the choriocapillaris (CC)

CC	Group I	Group II	*P* value
Whole image	65.135 ± 1.811	66.326 ± 1.678	0.105
Foveal VD	63.641 ± 3.619	62.377 ± 6.092	0.583
Parafoveal VD	64.823 ± 1.726	66.081 ± 1.845	0.106
Temporal quadrant VD	65.166 ± 1.863	66.739 ± 1.471	0.118
Superior quadrant VD	64.439 ± 2.180	65.386 ± 4.18	0.582
Nasal quadrant VD	64.957 ± 1.932	66.162 ± 3.062	0.694
Inferior quadrant VD	64.877 ± 2.130	66.073 ± 4.118	0.227

*cc: choriocapillaris, VD: vessel density

**Table 5:** The thickness of different zones

Thickness	Group I	Group II	*P* value
Fovea	238.5 ± 23.04	224.23 ± 16.61	0.185
Parafovea	307.75 ± 19.78	306.45 ± 14.54	0.35
Temporal quadrant	299.18 ± 16.34	301.32 ± 15.23	0.084
Superior quadrant	311.04 ± 17.74	309.14 ± 19.3	0.109
Nasal quadrant	309.82 ± 19.28	307.91 ± 18.87	0.109
Inferior quadrant	308.11 ± 21.74	308.83 ± 17.92	0.471

## Data Availability

No datasets were generated or analysed during the current study.

## Abbreviations

OCTA: Optical coherence tomography angiography
OCT: Optical coherence tomography
GH: Growth hormone
GHD: Growth hormone deficiency
IGHD: Isolated growth hormone deficiency
GHRH: Growth hormone-releasing hormone
IGF-1: Insulin-like growth factor-1
VD: Vessel density
SCP: Superficial capillary plexus
DCP: Deep capillary plexus
CC: Choriocapillaris
